# Liver Transaminases in Pediatric Adenovirus Infection—A Five-Year Study in Two Major Reference Centers from Romania

**DOI:** 10.3390/microorganisms11020302

**Published:** 2023-01-24

**Authors:** Oana Săndulescu, Anca Streinu-Cercel, Victor Daniel Miron, Silvia Mirela Covăcescu, Adrian Streinu-Cercel, Mihai Craiu

**Affiliations:** 1 Carol Davila University of Medicine and Pharmacy, 050474 Bucharest, Romania; 2 National Institute for Infectious Diseases “Prof. Dr. Matei Balș”, 021105 Bucharest, Romania; 3 National Institute of Mother and Child Health “Alessandrescu-Rusescu”, 020395 Bucharest, Romania

**Keywords:** human adenovirus, children, liver cytolysis, transaminases

## Abstract

Human adenovirus causes infections with a very heterogeneous clinical picture, and children are often the most frequently affected group. Interest in adenovirus has increased with the 2022 outbreak of severe acute hepatitis of unknown etiology as human adenovirus was considered as one of the possible etiological agents. We conducted a retrospective study over a 5-year period in two major tertiary hospitals in the Romanian capital with the aim to characterize the clinical picture and the dynamics of liver function tests in children with confirmed adenovirus infection. The study included 1416 children with a median age of 1.1 years (IQR: 0.3, 2.3 years). Digestive symptoms were predominant in 95.2% of children, mainly diarrhea (90.5%) and vomiting (50.5%), and 38.0% had respiratory symptoms. Increased transaminases were identified in 21.5% of patients. Age over 1 year, lethargy, vomiting and dehydration significantly increased the odds of liver cytolysis independent of other risk factors such as chronic conditions or co-infections. Aspartate aminotransferase (AST) was more commonly increased compared to alanine aminotransferase (ALT). Only six children had transaminase increases above 500 U/L, three of which had co-infections with rotavirus, Epstein–Barr virus (EBV), or respiratory syncytial virus (RSV). Liver function tests should be part of routine monitoring for pediatric patients with adenovirus infection. The current study fills a gap in current knowledge related to the frequency and the extent of liver involvement in human adenovirus infection among pediatric patients.

## 1. Introduction

Since April 2022, human adenovirus (HAdV) infection among children has become the focus of worldwide medical attention with alarm signals coming from the United Kingdom about a possible association between HAdV and the outbreak of severe acute hepatitis in children [1,2,3]. However, HAdV has been known for over 69 years [4], causing a wide spectrum of acute illnesses, with manifestations varying from digestive and respiratory to genitourinary or ocular [5]. So far, over 110 types and genotypes of HAdV have been described in human pathology [6]. Although no clear seasonality has been described and HAdV infections can occur throughout the year, outbreaks can appear during cold seasons [7,8,9]. The incidence of HAdV infections in children is higher than in adults, leading to an increased number of hospitalizations in the pediatric population and an increased burden on healthcare systems [10].

Due to their specific biological characteristics, adenoviruses are used as vectors in the development of vaccines and gene therapies for other infectious diseases or neoplastic diseases [11,12]. Although extensive research is underway, a vaccine to prevent adenovirus infections is not yet widely available. In the USA, an adenovirus vaccine containing live-attenuated HAdV types 4 and 7 is recommended for military personnel [13].

Severe hepatic involvement in HAdV infection was reported prior to the 2022 outbreak, with a number of case reports published in the literature [14], most of them occurring in immunosuppressed patients [15,16,17,18]. Overall, the dynamics of laboratory parameters in HAdV infection are insufficiently quantified, with most published data referring only to severe cases. In this context, we aimed to analyze the changes occurring in the dynamics of liver function tests, specifically transaminases, in children with HAdV infection regardless of disease severity in relation to their demographic and clinical characteristics to ensure a better understanding of the natural evolution of the disease.

## 2. Materials and Methods

We conducted a retrospective study among children hospitalized with confirmed a HAdV infection between 1 January 2017 and 31 December 2021. Children were selected from two tertiary hospitals in Bucharest, the capital of Romania: the National Institute for Infectious Diseases “Prof. Dr. Matei Balș” (NIID) and the National Institute for Mother and Child Health “Alessandrescu-Rusescu” (NIMCH). The NIID is the biggest tertiary infectious disease hospital in Romania and the NIMCH is an important tertiary pediatric hospital serving the northern area of the capital together with the whole adjacent metropolitan area.

In the pre-pandemic period, children diagnosed with adenovirus enterocolitis with ages over 1 year old were hospitalized in the NIID and infants (children under 1 year of age) in the NIMCH. For cases of adenovirus with other primary sites of infection, there were no specific regulations. During the first two years of the pandemic (March 2020 and throughout 2021), the NIID became a COVID-19-only hospital, and hospitalizations for all children with adenovirus infection, regardless of age, occurred in the NIMCH. Hospitalizations for non-COVID-19 infectious diseases were resumed in the NIID in 2022. For this reason, we chose to analyze the cases in both hospitals in order to provide a complete picture throughout the entire pediatric age range and the entire study period.

For this study, we retrieved from the two hospitals all cases that had ICD-10 diagnosis codes: A08.2, A85.1, A87.1, B30.0, B30.1, B34.0, B97.0, and J12.0. Each case was analyzed by two of the authors of this article. All patients hospitalized during the study period that were under 18 years of age, showed evidence of adenovirus infection (rapid stool antigen test, rapid nasopharyngeal swab antigen test, multiplex RT-PCR from stool or nasopharyngeal swab), and had clinical and laboratory data (transaminase values) available were included in the study. Outpatients and those with incomplete data were excluded. We recorded all data throughout the course of hospital admission for each confirmed case. If there were multiple transaminase determinations, we recorded the highest value.

The identification of adenovirus etiology differed slightly in the two hospitals over the 5 years included in the analysis. In the pre-pandemic period in the NIMCH, identification of adenovirus was only possible by rapid stool antigen tests, and since April 2020, this has been done by rapid stool or nasopharyngeal swab antigen tests and multiplex RT-PCR from nasopharyngeal swab. In the NIID, adenovirus was identified by rapid antigen tests from stool and multiplex RT-PCR from stool or nasopharyngeal swab both before and during the pandemic.

A child with HAdV infection was considered to have digestive manifestations if at least one of the following symptoms was present: diarrhea, vomiting, nausea, acholic stool, blood in stool (bloody diarrhea), or abdominal pain. Respiratory manifestations were defined as the presence of at least one of the following symptoms: cough, dyspnea, rhinorrhea, or nasal obstruction. Abdominal pain and nausea were quantified only in children over 2 years of age.

Increased transaminases were defined as any value above the upper limit of the normal range for age and sex according to the local laboratories of the two hospitals (Supplementary Material). Identification of other pathogens in the stool sample or the respiratory sample was classified as co-infection. Pathogens identified in urine culture were excluded from the co-infection analysis. 

Statistical analysis of the data was performed using IBM SPSS Statistics for Windows, version 25 (IBM Corp., Armonk, NY, USA). Any value of *p* < 0.05 was considered statistically significant. In the analysis of the qualitative variables, we used the Chi-square test, and we report the result together with the odds ratio (OR) and confidence interval (95% CI). Because all our continuous variables had non-Gaussian distribution, we report the median with interquartile range (IQR) (25th–75th percentile) and Mann–Whitney U test results for comparison.

## 3. Results

### 3.1. Demographic and Clinical Characteristics

**Figure 1:**
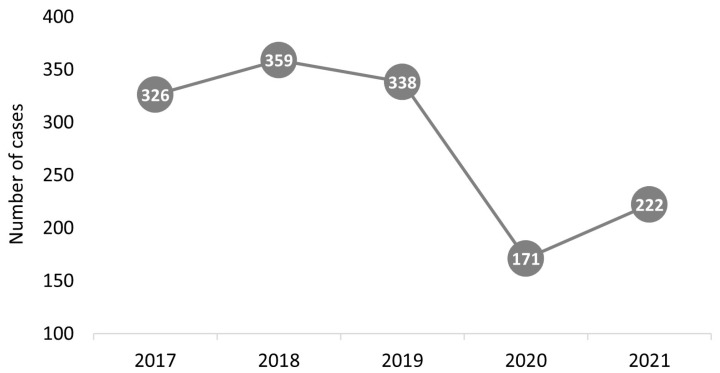
Distribution of pediatric adenovirus cases per year.

A total of 1416 patients with confirmed HAdV infection were included in the study. The median age was 1.1 years (IQR: 0.3, 2.3 years), with a male predominance (56.5%, *n* = 800). There was no age difference between the sexes (1 year (IQR: 0.3, 2.3 years) for female patients and 1.1 years (IQR: 0.3, 2.3 years) for male patients, *p* = 0.958). Most cases were from the pre-pandemic period, with the distribution of cases by year shown in Figure 1. We did not identify any outbreaks in the 5 years studied, with cases occurring evenly throughout the seasons covered.

HAdV was identified in 98.7% (*n* = 1397) of cases by rapid stool antigen test, in only 5.7% (*n* = 81) of cases by rapid nasopharyngeal swab antigen test, and in 0.7% (*n* = 10) by nasopharyngeal swab multiplex RT-PCR, respectively.

**Figure 2:**
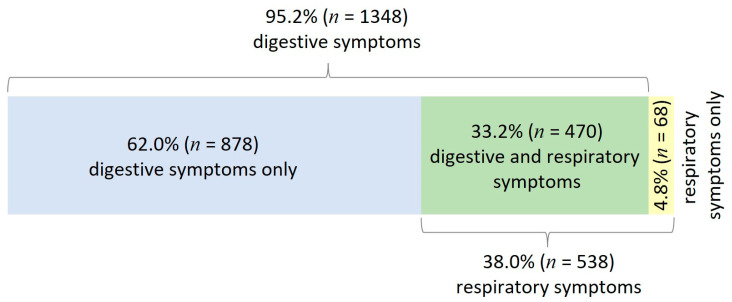
Distribution of symptoms in pediatric patients with adenovirus infection.

Digestive symptoms were present in the majority of children (95.2%, *n* = 1348), 33.2% (*n* = 470) also associated respiratory symptoms, and 4.8% (*n* = 68) had respiratory symptoms only (Figure 2). Overall, the most common symptoms were diarrhea (90.5%, *n* = 1281), fever (55.4%, *n* = 785) and vomiting (50.5%, *n* = 715) (Table 1). Only 3.8% (*n* = 54) of the children included in the study had a chronic condition, the most common being neurological disease and cystic fibrosis (*n* = 15/54, 27.7% for each, Table 1). Co-infections increased the risk of fever (1.8-fold), blood in stool (1.9-fold), dehydration (1.8-fold), and higher C-reactive protein values (1.5-fold) (Table 1).

**Figure 3:**
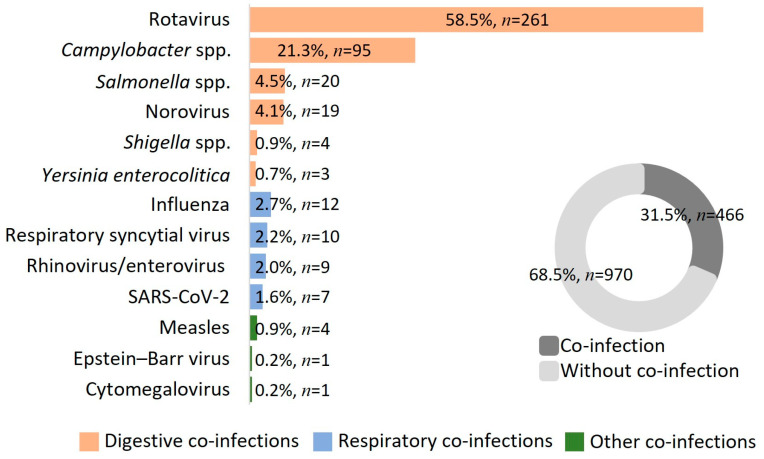
Distribution of co-infections identified in pediatric patients with adenovirus infection.

Co-infections were found in 31.5% (*n* = 446) of children. Digestive pathogens (86.3%, *n* = 402) such as rotavirus (58.5% *n* = 261), *Campylobacter* spp. (6.7% *n* = 95), and *Salmonella* spp. (4.5%, *n* = 20) were the most frequently identified. There were also non-digestive co-infections with influenza viruses, SARS-CoV-2, measles, or Epstein–Barr virus (EBV) (Figure 3).

The median time from symptom onset to hospital presentation was 2 days (IQR: 1, 3 days). It was not influenced by the child’s sex, age group (infant/non-infant), presence of chronic condition, or co-infection (*p* > 0.05 for each). The median length of hospital stay was 4 days (IQR: 3, 6 days). This was not influenced by the patient’s sex but was higher among infants (5 days (IQR: 3, 7 days) compared to 4 days (IQR: 3, 5 days) for non-infants, *p* < 0.001, z = −9.0, r = 0.24). The presence of a chronic condition prolonged hospitalization to 6.5 days (IQR: 5, 11.8 days) (*p* < 0.001, U = 25761, z = −5.85, r = 0.16) and the presence of a co-infection, to 5 days (IQR: 3, 7 days) (*p* < 0.001, U = 172263.5, z = −6.22, r = 0.17).

### 3.2. Analysis of Liver Function Tests

**Figure 4:**
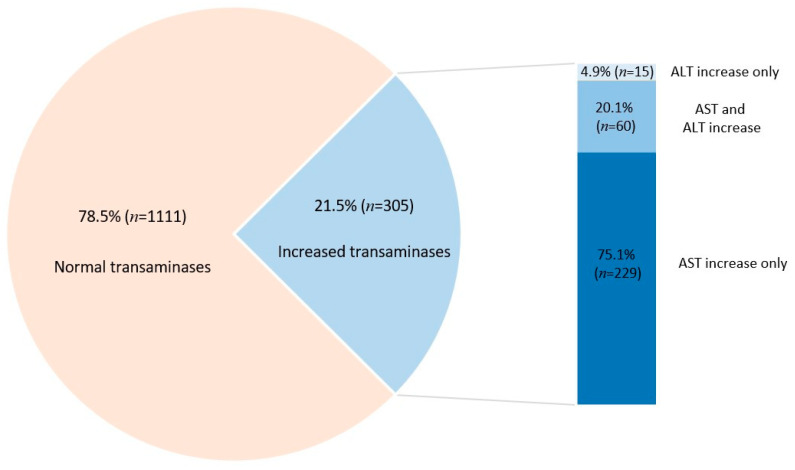
Distribution of transaminases values in pediatric patients with adenovirus infection.

We identified transaminase values above the normal range in 21.5% (*n* = 305) of the children; among these, 15 (4.9%) had increased alanine aminotransferase (ALT) only, 229 (75.1%) had increased aspartate aminotransferase (AST) only, and 61 (20%) displayed increases in both transaminase values (Figure 4). Overall, the median value for ALT was 29 U/L (IQR: 22, 38 U/L) and for AST, 45 U/L (IQR: 36, 59 U/L).

Children older than 1 year had a 3.0-fold higher risk of increased transaminases (*p* < 0.001). Clinically, lethargy, vomiting and dehydration increased the odds of presenting liver cytolysis by 2.9-, 1.9-, and 1.9-fold, respectively (*p* < 0.05 for each, Table 2). In contrast, the presence of blood in the stool, *Campylobacter* co-infection, and increased serum C-reactive protein values were associated with lower odds of cytolysis. Comparisons between the two groups are highlighted in Table 2. 

Among the transaminases, increased AST was associated with age over 1 year (OR = 4.2, *p* < 0.001) and the presence of digestive symptoms (OR = 5.3, *p* = 0.006), especially diarrhea (OR = 3.0, *p* = 0.007) and vomiting (OR = 3.1, *p* < 0.001), Table 3. A concomitant increase in ALT and AST was found more often in patients with chronic conditions (OR = 6.9, *p* = 0.001) and in those with co-infections (OR = 2.2, *p* = 0.008), especially rotavirus co-infection (OR = 2.3, *p* = 0.009), Table 3.

The number of days of illness prior to hospital admission were not associated with increased transaminases, median values being identical to those for the whole studied group. Regardless of the value or the presence or absence of liver cytolysis, the median length of hospitalization was similar (4 days (IQR: 3, 6 days), *p* = 0.350), but in the subgroup analysis, only the increase in ALT resulted in increased length of hospitalization (5 days (IQR: 4, 11 days), *p* < 0.001).

After excluding patients with chronic conditions and those with co-infections, the following factors: age over 1 year, lethargy, vomiting, and dehydration remained prognostic factors for increased transaminases, with increasing OR for each (Table 4). In addition, the presence of diarrhea was shown to increase the risk of liver cytolysis 2.4-fold (OR = 2.4, *p* = 0.010) (Table 4). In this exclusion analysis, increased AST remained associated with age over 1 year (OR = 6.5, *p* < 0.001) and vomiting (OR = 3.9, *p* = 0.001), with increasing ORs (Table 5). The number of days of illness prior to hospital presentation or the length of hospital stay were not significantly influenced in this case (*p* > 0.05 in all cases).

Values above 500 U/L of transaminases, as those used for the definition of severe acute hepatitis in children during the 2022 outbreak, were observed in six cases. Their characteristics are summarized in Table 6. All cases had a favorable outcome, but case 1 required admission to intensive care, being a child with congenital heart disease.

## 4. Discussion

The biology of adenoviruses is complex, and the clinical manifestations of HAdV infection are polymorphic, from asymptomatic and mild to severe forms with unfavorable outcomes. Following the report by Marsh et al. [1] in April 2022 in which thirteen cases of acute severe hepatitis of unknown etiology (AS-Hep-UA) were described, interest in monitoring HAdV infections has increased significantly, as in five of these cases, HAdV was identified as the possible etiology. In the latest joint report by the European Centre for Disease Prevention and Control and World Health Organization Regional Office for Europe on HUE, the positivity rate for HAdV was 51.6% out of 457 probable cases tested [19]. Overall globally, 46.7% of samples tested for HAdV were positive, and of these, adenovirus type 41 was the most common [20]. However, there is no clear evidence to confirm that HAdV is the etiological agent responsible for the AS-Hep-UA outbreak [21,22]. As mentioned in the introduction, a number of pediatric cases of adenovirus-associated hepatitis, including adenovirus type 41, have been published over time, but these have been reported in immunocompromised children [14,23,24,25]. Recent reports show the involvement of HAdV as a possible etiology in adults with AS-Hep-UA [26].

Data on the clinical impact and degree of liver cytolysis induced by HAdV infection in children with non-severe forms of the disease are limited. Therefore, the main aim of our study was to evaluate the dynamics of clinical manifestations and laboratory changes of transaminases in hospitalized children with confirmed HAdV infection, across the entire spectrum of illness severity, over 5 years in two tertiary hospitals in the Romanian capital. We retrospectively collected clinical data, taking into account the case definition for AS-Hep-UA [21] so as to provide as clear a picture as possible of HAdV infection that can be used for further analysis.

The majority of cases included in the analysis were from the pre-pandemic period, which can be explained by the fact that viral circulation and hospitalizations in the pediatric population were significantly influenced by the COVID-19 pandemic and the temporary prevention measures implemented [27,28,29]. We identified that digestive symptoms (95.2%) were predominant in most children, especially diarrhea (90.5%) and vomiting (50.5%). It is known that second to rotavirus, adenovirus is one of the main viral agents of enterocolitis in children [10]. Rotavirus co-infection was commonly found in our study in 261 of the children (58.5% of those with co-infection). This percentage is much higher than previously reported [30,31,32], but this can be explained by the fact that the rotavirus vaccine is optional in our country, and the uptake rate is low [33]. 

More than 80% of HAdV infections occur in children younger than 4 years old [34] and account for 5–10% of pediatric respiratory infections [35,36]. Respiratory symptoms, isolated or concomitant with digestive symptoms (4.8% and 33.2% in our study, respectively) are common in adenoviral infections in children. These features have been documented even during the SARS-CoV-2 pandemic [37]. In Romania, 14.2% of bronchiolitis cases that were not attributable to RSV or human metapneumovirus were generated by HAdV [38]. We also identified co-infections with respiratory viruses such as influenza, rhinovirus, and SARS-CoV-2. These types of co-infections have become increasingly evident with the introduction of multiplex RT-PCR testing, but also with the existence of rapid antigenic tests for HAdV, which are also used in our country.

Overall, we identified increases in transaminases in 21.5% of children with HAdV, but only six children had increases above 500 U/L. Lethargy was a clinical indicator of the presence of hepatic cytolysis in our study. Patients with acute viral hepatitis may show a high degree of fatigue and lethargy, which may be related to the severity of liver disease [39,40]. The direct association of cytolysis with vomiting and dehydration identified by us has not been clearly described to date, especially for cases of HAdV infection. These two clinical manifestations are part of the clinical picture of acute hepatitis of any cause, but their link with HAdV has not been evidenced so far. Increases in AST were more common than increases in ALT, being significantly associated with age over 1 year and the presence of digestive symptoms. Co-infection and, in particular, rotavirus co-infection led to an increase in both transaminases. Kucuk et al. [41] previously showed that ALT increases are more common in rotavirus compared to adenovirus enterocolitis. In contrast, co-infection with *Campylobacter* spp. was a protective factor for liver cytolysis, being more common among patients with normal transaminase values. We have not identified a clear pathophysiological explanation for this finding, given that cases of hepatitis associated with *Campylobacter* enteritis have been reported [42,43,44]. To this aim, further studies are needed to explore the interactions between the two infectious agents and the impact on liver damage. C-reactive protein was shown to be inversely associated with transaminase values. However, it should be noted that when patients with chronic conditions and co-infections were excluded from the analysis, this association was no longer observed.

Of the six children with transaminases above 500 U/L, three had other co-infections such as rotavirus, EBV, or RSV. Cases of hepatitis associated with rotavirus infection have been reported in the literature, including in immunocompetent children [45,46]. Hepatic involvement in EBV infection is well-known and data on pathophysiological mechanisms are clear [47]. RSV has also been recognized to be involved in extra-respiratory manifestations and to cause hepatic cytolysis, especially among young children [48,49]. In the other three children, other etiologies were excluded, and HAdV remained the only etiologic agent identified. Of note is the case of the 6-month-old infant with congenital heart disease who presented only respiratory manifestations and required monitoring in the intensive care unit. This case highlights that transaminase monitoring is absolutely necessary even in infants with respiratory HAdV.

The present study has a number of limitations. The retrospective nature of the study may have predisposed to bias in interpretation and the classification of data, and it was not possible to follow patients over longer periods of time. In addition, the methods of testing and identification of HAdV varied across the study period; therefore, there may be a certain degree of case selection bias. There were also no data on the type of HAdV. Nevertheless, we included in the study a large number of patients from two major hospitals in Romania and we extensively characterized cases across the entire spectrum of illness severity. Thus, to the best of our knowledge, this study provides a comprehensive overview of the clinical picture and the dynamics of transaminases in children with HAdV in the current context of increased interest in these viruses.

## 5. Conclusions

Increased transaminases in children with HAdV infection were identified in 21.5% of patients, but marked increases above 500 U/L were rare (six cases) and were associated with other co-infections (in three of these cases). HAdV infection has a polymorphic clinical presentation, and transaminase monitoring should be part of routine testing for a pediatric patient with HAdV infection. Age over 1 year, lethargy, and the presence of digestive manifestations were predictive factors for liver cytolysis in children with adenovirus infection.

## Figures and Tables

**Table 1:** Characteristics of the studied group.

Characteristics	Total, *n* = 1416, *n* (%)	Adenovirus Infection Only, *n* = 970, *n* (%)	Adenovirus with Co-Infection, *n* = 446, *n* (%)	*p*-Value
**Demographic data**				
Male sex	800 (56.5)	551 (56.8)	249 (55.8)	0.731
Female sex	616 (43.5)	419 (43.2)	197 (44.2)	
Infants	701 (49.5)	454 (46.8)	247 (55.4)	0.003, OR = 0.7, 95% CI: 0.6–0.8
Non-infants (over 1 year)	715 (50.5)	516 (53.2)	199 (44.6)	
**Symptoms**				
Lethargy	626 (44.2)	438 (45.2)	188 (42.2)	0.291
Fever	785 (55.4)	494 (50.9)	291 (65.2)	<0.001, OR = 1.8, 95% CI: 1.4–2.3
Digestive symptoms	1348 (95.2)	921 (94.9)	427 (95.7)	0.518
Abdominal pain *	116/462 (25.1)	92/351 (26.2)	24/111 (21.6)	0.332
Diarrhea	1281 (90.5)	875 (90.2)	406 (91.0)	0.623
Blood in stool	157 (11.1)	86 (8.9)	71 (15.9)	<0.001, OR = 1.9, 95% CI: 1.4–2.7
Acholic stool	0 (0.0)	0 (0.0)	0 (0.0)	NA
Nausea *	21/462 (4.5)	17/351 (4.8)	4/111 (3.6)	0.583
Vomiting	715 (50.5)	508 (52.4)	207 (46.4)	0.052
Respiratory symptoms **	538 (38.0)	370 (38.1)	168 (37.7)	0.864
Rash	117 (8.3)	85 (8.8)	32 (7.2)	0.313
Conjunctivitis	36 (2.5)	27 (2.8)	9 (2.0)	0.395
Jaundice ***	0 (0.0)	0 (0.0)	0 (0.0)	NA
**Chronic conditions**				
At least one chronic disease	54 (3.8)	47 (4.8)	7 (1.7)	0.002, OR = 3.2, 95% CI: 1.4–7.1
Neurological diseases	15 (0.9)	13 (1.3)	2 (0.4)	0.134
Genetic syndromes	9 (0.6)	8 (0.8)	1 (0.2)	0.254
Cystic fibrosis	15 (1.1)	11 (1.1)	4 (0.9)	0.604
Renal diseases	10 (0.7)	10 (1.0)	0 (0.0)	NA
Heart malformations	1 (0.1)	1 (0.1)	0 (0.0)	NA
Immune diseases	1 (0.1)	1 (0.1)	0 (0.0)	NA
Tuberculosis	2 (0.2)	2 (0.2)	0 (0.0)	NA
Leukemia	1 (0.1)	1 (0.1)	0 (0.0)	NA
**Dehydration**	1206 (85.2)	805 (83.0)	401 (89.9)	0.001, OR = 1.8, 95% CI: 1.3–2.6
**Increased C-reactive protein**	667 (47.1)	393 (43.8)	225 (54.5)	<0.001, OR = 1.5, 95% CI: 1.2–1.9

* Only for children over 2 years old (*n* = 462); ** cough and/or nasal obstruction and/or rhinorrhea and/or dyspnea; *** newborns with physiological jaundice were excluded; NA—not applicable.

**Table 2:** Predictors of increased liver enzymes.

Characteristics	Increased Transaminases, *n* = 305, *n* (%)	Normal Transaminases, *n* = 1111, *n* (%)	Statistical Analysis
Male sex	161 (52.8)	639 (57.5)	*p* = 0.140
Non-infants	217 (71.1) ^#^	498 (44.8)	*p* < 0.001, χ^2^ = 66.3, OR = 3.0, 95% CI: 2.3–4.0
Lethargy	197(64.6) ^#^	429 (38.6)	*p* = 0.001, χ^2^ = 65.5, OR = 2.9, 95% CI: 2.2–3.8
Fever	168 (55.1)	617 (55.5)	*p* = 0.888
Digestive symptoms	292 (95.7)	1056 (95.0)	*p* = 0.619
Abdominal pain *	28/125 (22.4)	88/337 (26.1)	*p* = 0.413
Diarrhea	275 (90.2)	1006 (90.5)	*p* = 0.839
Blood in stool	12 (3.9)	145 (13.1) ^#^	*p* < 0.001, χ^2^ = 20.2, OR = 0.2, 95% CI: 0.1–0.5
Nausea *	5/125 (4.0)	16/337 (4.7)	*p* = 0.729
Vomiting	191 (62.6) ^#^	524 (47.2)	*p* < 0.001, χ^2^ = 22.9, OR = 1.9, 95% CI: 1.4–2.4
Respiratory symptoms	119 (39.0)	420 (37.8)	*p* = 0.942
Rash	28 (9.2)	89 (8.0)	*p* = 0.511
Conjunctivitis	4 (1.3)	32 (2.9)	*p* = 0.123
Digestive and respiratory symptoms	106 (34.8)	364 (32.8)	*p* = 0.513
Other chronic illnesses	15 (4.9)	39 (3.5)	*p* = 0.256
Co-infections	104 (34.1)	342 (30.8)	*p* = 0.270
Rotavirus	67 (22.0)	194 (17.5)	*p* = 0.072
Norovirus	5 (1.6)	14	*p* = 0.610
Campylobacter	9 (3.0)	86 (7.7) ^#^	*p* = 0.003, χ^2^ = 8.7, OR = 0.4, 95% CI: 0.2–0.7
Other co-infections	21 (7.5)	48 (4.3)	*p* = 0.065
Dehydration	276 (90.5) ^#^	930 (83.7)	*p* = 0.003, χ^2^ = 8.7, OR = 1.9, 95% CI: 1.2–2.8
Increased C-reactive protein	126 (41.3)	541 (48.7) ^#^	*p* = 0.033, χ^2^ = 4.5, OR = 0.7, 95% CI: 0.5–0.9

* Only for children over 2 years old (*n* = 462); ^#^ group with statistical significance; NA—not applicable.

**Table 3:** Comparative analysis according to transaminase type.

Characteristics	ALT Increase Only, *n* = 15, *n* (%)	AST Increase Only, *n* = 229, *n* (%)	ALT and AST Increase, *n* = 61, *n* (%)	Statistical Analysis
Male sex	4 (26.7)	127 (55.5)	30 (49.2)	*p* = 0.079
Non-infants	3 (20.0)	181 (79.0) ^#^	33 (54.1)	*p* < 0.001, χ^2^ = 27.9, OR = 4.2, 95% CI: 2.4–7.3
Lethargy	6 (40.0)	158 (69.0)	33 (54.1)	*p* = 0.012
Fever	9 (60.0)	121 (52.8)	38 (62.3)	*p* = 0.388
Digestive symptoms	13 (86.7)	224 (97.8) ^#^	55 (90.2)	*p* = 0.006, χ^2^ = 7.2, OR = 5.3, 95% CI: 1.7–16.6
Abdominal pain *	0/3 (0.0)	22/101 (21.8)	6/21 (28.8)	*p* = 0.371
Diarrhea	11 (73.3)	213 (93.0) ^#^	51 (83.6)	*p* = 0.007, χ^2^ = 8.4, OR = 3.0, 95% CI: 1.4–6.5
Blood in stool	2 (13.3)	8 (3.5)	2 (3.3)	*p* = 0.105
Nausea *	0/3 (0.0)	3/101 (3.0)	2/21 (9.5)	*p* = 0.776
Vomiting	2 (13.3)	159 (69.4) ^#^	30 (49.2)	*p* < 0.001, χ^2^ = 18.2, OR = 3.1, 95% CI: 1.8–5.3
Respiratory symptoms	8 (53.3)	81 (35.4)	27 (44.3)	*p* = 0.204
Rash	1 (6.7)	19 (8.3)	8 (13.1)	*p* = 0.482
Conjunctivitis	0 (0.0)	4 (1.7)	0 (0.0)	NA
Digestive and respiratory symptoms	6 (40.0)	79 (34.5)	21 (34.4)	*p* = 0.909
Other chronic illnesses	1 (6.6)	5 (2.2)	9 (14.8) ^#^	*p* = 0.001, χ^2^ = 9.7, OR = 6.9, 95% CI: 2.3–20.1
Co-infections	7 (46.7)	67 (29.3)	30 (49.2) ^#^	*p* = 0.008, χ^2^ = 7.7, OR = 2.2, 95% CI: 1.3–3.9
Rotavirus	3 (20.0)	43 (18.8)	21 (34.4) ^#^	*p* = 0.009, χ^2^ = 6.9, OR = 2.3, 95% CI: 1.2–4.2
Norovirus	0 (0.0)	5 (2.2)	0 (0.0)	NA
Campylobacter	1 (6.7)	6 (2.6)	2 (3.3)	*p* = 0.659
Other co-infections	2 (13.3)	13 (5.7)	6 (9.8)	*p* = 0.254
Dehydration	13 (86.7)	210 (91.7)	53 (86.9)	*p* = 0.457
Increased C-reactive protein	8 (53.3)	87 (38.0)	31 (50.8)	*p* = 0.180

* Only for children over 2 years old (*n* = 125); ^#^ group with statistical significance; NA—not applicable.

**Table 4:** Comparative analysis in relation to transaminase increase after exclusion of patients with chronic conditions and co-infections.

Characteristics	Increased Transaminases, *n* = 194, *n* (%)	Normal Transaminases, *n* = 729, *n* (%)	Statistical Analysis
Male sex	112 (57.7)	416/729 (57.1)	*p* = 0.867
Non-infants	149 (76.8) ^#^	350 (48.0)	*p* < 0.001, χ^2^ = 51.2, OR = 3.6, 95% CI: 2.5–5.2
Lethargy	130 (67.0) ^#^	291 (39.9)	*p* < 0.001, χ^2^ = 45.3, OR = 3.0, 95% CI: 2.2–4.3
Fever	98 (50.5)	372 (51.0)	*p* = 0.899
Digestive symptoms	190 (97.9)	689 (94.5)	*p* = 0.057
Abdominal pain *	16/88 (18.2)	63/249 (25.3)	*p* = 0.174
Diarrhea	185 (95.4) ^#^	651 (89.3)	*p* = 0.010, χ^2^ = 6.6, OR = 2.4, 95% CI: 1.2–5.0
Blood in stool	6 (3.1)	74 (10.2) ^#^	*p* = 0.002, χ^2^ = 9.6, OR = 0.3, 95% CI: 0.1–0.7
Nausea *	4/88 (4.5)	13/249 (5.2)	*p* = 0.619
Vomiting	136 (70.1) ^#^	355 (48.7)	*p* < 0.001, χ^2^ = 28.2, OR = 2.4, 95% CI: 1.8–3.4
Respiratory symptoms	66 (34.0)	283 (38.8)	*p* = 0.220
Rash	19 (9.8)	61 (8.4)	*p* = 0.530
Conjunctivitis	3 (1.5)	23 (3.2)	*p* = 0.229
Digestive and respiratory symptoms	62 (32.0)	243 (33.3)	*p* = 0.718
Dehydration	178 (91.8) ^#^	591 (81.1)	*p* < 0.001, χ^2^ = 12.6, OR = 2.6, 95% CI: 1.5–4.5
Increased C-reactive protein	63 (37.3)	310 (45.3)	*p* = 0.059

* Only for children over 2 years old (*n* = 337); ^#^ group with statistical significance; NA—not applicable.

**Table 5:** Comparative analysis according to transaminase type after exclusion of patients with chronic conditions and co-infections.

Characteristics	ALT Increase Only, *n* = 8, *n* (%)	AST Increase Only, *n* = 160, *n* (%)	ALT and AST Increase, *n* = 26, *n* (%)	*p*-Value
Male sex	1 (12.5)	97 (58.5)	17 (65.4)	0.025
Non-infants	1 (12.5)	134 (83.8) ^#^	14 (53.8)	*p* < 0.001, χ^2^ = 24.7, OR = 6.5, 95% CI: 2.9–14.5
Lethargy	1 (12.5)	117 (73.1)	12 (46.2)	<0.001
Fever	3 (37.5)	78 (48.8)	17 (65.4)	0.219
Digestive symptoms	8 (100)	157 (98.1)	25 (96.2)	0.739
Abdominal pain *	0/1 (0.0)	14/78 (17.9)	2/9 (22.2)	0.231
Diarrhea	8 (100)	153 (95.6)	24 (92.3)	0.618
Blood in stool	2 (25.0)	4 (2.5)	0 (0.0)	NA
Nausea *	0/1 (0.0)	3/78 (3.8)	1/9 (11.1)	0.259
Vomiting	2 (25.0)	121 (75.6) ^#^	13 (50.0)	*p* = 0.001, χ^2^ = 13.3, OR = 3.9, 95% CI: 1.8–8.5
Respiratory symptoms	4 (50.0)	52 (35.2)	10 (35.8)	0.521
Rash	0 (0.0)	15 (9.4)	4 (15.4)	0.402
Conjunctivitis	0 (0.0)	3 (1.9)	0 (0.0)	NA
Digestive and respiratory symptoms	4 (50.0)	49 (30.6)	9 (34.6)	0.493
Dehydration	7 (87.5)	148 (92.5)	23 (88.5)	0.711
Increased C-reactive protein	3 (37.5)	49 (36.0)	11 (44.0)	0.751

* Only for children over 2 years old (*n* = 88); ^#^ group with statistical significance; NA—not applicable.

**Table 6:** Characteristics of cases with transaminase values above 500 U/L.

	Case 1	Case 2	Case 3	Case 4	Case 5	Case 6
Sex	Female	Female	Male	Female	Male	Female
Age	6 months	3.3 years	2.3 years	8.6 years	10 months	4 months
Symptoms	Lethargy, fever, cough, rhinorrhea, shortness of breath	Fever, diarrhea, abdominal pain, vomiting	Lethargy, fever, diarrhea, vomiting	Fever, abdominal pain, vomiting, nasal obstruction	Diarrhea, cough, nasal obstruction	Cough, rhinorrhea, dyspnea
Days since onset of symptoms	3 days	1 day	2 days	5 days	2 days	1 day
Days of hospitalization	32 days	8 days	4 days	5 days	10 days	12 days
ALT value	1116 U/L	1053 U/L	739 U/L	2200 U/L	629 U/L	985 U/L
AST value	802 U/L	961 U/L	340 U/L	1482 U/L	356 U/L	764 U/L
Co-infection	No	Rotavirus	No	EBV	No	RSV

AST—aspartate aminotransferase; ALT—alanine aminotransferase; EBV—Epstein–Barr virus; RSV—respiratory syncytial virus.

## Data Availability

The anonymized data presented in this study are available upon reasonable request from the corresponding author.

## Supplementary Materials

The following supporting information can be downloaded at: https://www.mdpi.com/article/10.3390/microorganisms11020302/s1, Table S1: Normal ranges of transaminases.
