# Supplementary material for: Liver Transaminases in Pediatric Adenovirus Infection—A Five-Year Study in Two Major Reference Centers from Romania
Source: Microorganisms. 2023 Jan 24;11(2):302. doi: 10.3390/microorganisms11020302 (PMC9961354; doi:10.3390/microorganisms11020302)
Supplement: Supplementary file 1 [file microorganisms-11-00302-s001.zip › microorganisms-2168644-supplementary.pdf]

Supplementary material

Normal ranges of transaminases

National Institute for Infectious Diseases ″Prof. Dr. Matei Balș″, Bucharest, Romania

Age	AST normal range	ALT normal range
Under 1 year	5 – 50 U/L	5 – 65 U/L
Over 1 year	10 – 37 U/L	10 – 60 U/L

National Institute of Mother and Child Health ″Alessandrescu-Rusescu″, Bucharest, Romania

Age	AST normal range	ALT normal range
Under 1 year	6 – 56 U/L	6 – 60 U/L
Over 1 year	7 – 45 U/L	7 – 55 U/L
